# Genes Encoding the Virulence and the Antimicrobial Resistance in Enterotoxigenic and Shiga-toxigenic *E. coli* Isolated from Diarrheic Calves

**DOI:** 10.3390/toxins12060383

**Published:** 2020-06-10

**Authors:** Abdelazeem M. Algammal, Ali W. El-Kholy, Emad M. Riad, Hossam E. Mohamed, Mahmoud M. Elhaig, Sulaiman A. Al Yousef, Wael N. Hozzein, Madeha O. I. Ghobashy

**Affiliations:** 1 Department of Bacteriology, Immunology and Mycology, Faculty of Veterinary Medicine, Suez Canal University, Ismailia 41522, Egypt; dr_aly_w@yahoo.com; 2 Department of Bacteriology, Animal Health Research Institute, Dokki, Giza 12618, Egypt; dremad8@yahoo.com (E.M.R.); drhossam199210@gmail.com (H.E.M.); 3 Department of Animal Medicine (Infectious Diseases), Faculty of Veterinary Medicine, Suez Canal University, Ismailia 41522, Egypt; melhaig@vet.suez.edu.eg; 4 Clinical Laboratories Sciences Department, College of Applied Medical Sciences, Hafr Albatin University, Hafr Al Batin 31991, Saudi Arabia; drsulaiman@uhb.edu.sa; 5 Bioproducts Research Chair, Zoology Department, College of Science, King Saud University, Riyadh 11451, Saudi Arabia; whozzein@ksu.edu.sa; 6 Botany and Microbiology Department, Faculty of Science, Beni-Suef University, Beni-Suef 62511, Egypt; 7 Microbiology Department, Faculty of Science, Ain Shams University-Cairo- Egypt, Cairo 11556, Egypt; Mghobashy@sci.asu.edu.eg

**Keywords:** ETEC, STEC, diarrhea, calves, virulence genes, antimicrobial resistance genes

## Abstract

Calf diarrhea is one of the considerable infectious diseases in calves, which results in tremendous economic losses globally. To determine the prevalence of Shiga-toxigenic *E. coli* (STEC) and Enterotoxigenic *E. coli* (ETEC) incriminated in calf diarrhea, with special reference to Shiga- toxins genes (*stx*1 and *stx*2) and enterotoxins genes (*lt* and *sta*) that govern their pathogenesis, as well as the virulence genes; *eae*A (intimin) and *f*41(fimbrial adhesion), and the screening of their antibiogram and antimicrobial resistance genes; *aad*B, *sul*1, and *bla-*TEM, a total of 274 fecal samples were collected (April 2018–Feb 2019) from diarrheic calves at different farms in El-Sharqia Governorate, Egypt. The bacteriological examination revealed that the prevalence of *E. coli* in diarrheic calves was 28.8%. The serotyping of the isolated *E. coli* revealed 7 serogroups; O_26_, O_128_, O_111_, O_125_, O_45_, O_119_ and O_91_. Furthermore, the Congo red binding test was carried out, where 89.8% of the examined strains (n = 71) were positive. The antibiogram of the isolated strains was investigated; the majority of *E. coli* serotypes exhibit multidrug resistance (MDR) to four antimicrobial agents; neomycin, gentamycin, streptomycin, and amikacin. Polymerase chain reaction (PCR) was used to detect the prevalence of the virulence genes; *stx*1, *stx*2 *lt*, *sta*, *f*41 and *eae*A, as well as the antimicrobial resistance genes; *aad*B, *sul*1, and *bla*-TEM. The prevalence of STEC was 20.2% (n = 16), while the prevalence of ETEC was 30.4% (n = 24). Briefly, the Shiga toxins genes; *stx*1 and *stx*2, are the most prevalent virulence genes associated with STEC, which are responsible for the pathogenesis of the disease and helped by the intimin gene (*eae*A). In addition, the *lt* gene is the most prevalent enterotoxin gene accompanied by the ETEC strains, either alone or in combination with *sta* and/or *f*41 genes. The majority of pathogenic *E. coli* incriminated in calf diarrhea possesses the *aad*B resistance gene, followed by the *sul*1 gene. Enrofloxacin, florfenicol, amoxicillin-clavulanic acid, and ampicillin-sulbactam, are the most effective antimicrobial agents against the isolated STEC and ETEC strains.

## 1. Introduction

Calf diarrhea is one of the most predominant syndromes in newly born animals all over the world, that are associated with remarkable economic losses, high morbidity and mortality rates [1]. The most prevalent bacterial pathogen which is incriminated in young calf’s diarrhea is *Escherichia. coli (E. coli*); moreover, the most common viral causes are *Rotavirus* and *Coronavirus* [2].

Based on the molecular and pathological criteria, the most common pathotypes of *E. coli* incriminated in neonatal colibacillosis are; Shiga-toxigenic *E. coli* (STEC), Enterotoxigenic *E. coli* (ETEC), and Enterohemorrhagic *E. coli* (EHEC) [3]. ETEC is a common pathotype associated with infectious diarrhea in calves. The newly born calves exhibit a high affinity to ETEC, which is associated with watery diarrhea. They colonize the small intestine after fimbrial adhesion and predispose to severe watery diarrhea [4]. Two main virulent factors are included; the fimbriae and the enterotoxins [5]. ETEC is skillful in generating 2 major types of enterotoxins; heat-labile (LT) and heat-stable (STa and STb) enterotoxins, in both man and animal [6].

Ruminants are considered the main reservoir of STEC; the severity of infection in ruminants varies depending upon the animal age, immunity, and the gastrointestinal tract conditions. Certain animals undergo the exaggerated shedding of STEC (>104 CFU/g fecal matter), which results in the contamination of the environment and the transmission of the infection [7]. A large number of STEC-outbreaks were reported, due to the ingestion of contaminated vegetables and fruits with the animal feces. STEC infection in humans is mainly associated with hemolytic uremic syndrome and hemorrhagic colitis. STEC has major public health importance, since it is incriminated in causing several food-borne outbreaks [8].

STEC are associated with dysentery in young calves. They produce two various potent types of Shiga-toxins; Stx1 and Stx2, and certain types of STEC have the ability to produce the intimin. The *E. coli* strains, which possess the *eae*A gene and do not produce *stx*1 and *stx*2 genes, were defined as Enteropathogenic *E. coli* [9,10]. The bacteriophages play a major role in the transmission of *stx* genes. The stx-phages are sharing an identical sequence that analogous to lambdoid-phages. The presence of *stx* genes in the phage lysis-portion illustrates the link between the production of Shiga-toxins and the release of phage during the lytic growth [3,10].

The antimicrobial resistance is usually associated with pathogenic *E. coli* that could be attributed to the widespread improper use of antibiotics. The multidrug resistance (MDR) is common in *E. coli* and primarily associated with several genes like; *bla*-TEM, *bla*CTX (β-lactamase genes), *sul1* (sulfonamide resistance gene), and *aad*B (aminoglycoside resistance gene) [11,12].

The current study was performed to investigate the prevalence of Shiga-toxigenic and Enterotoxigenic *E. coli* incriminated in calf diarrhea, with particular reference to Shiga-toxins genes (*stx*1 and *stx*2) and enterotoxins genes (*lt* and *sta*) that govern their pathogenesis, as well as the virulence genes; *eae*A and *f*41. In addition, the screening of their antibiogram and antimicrobial resistance genes; *aad*B (aminoglycosides-resistance gene), *sul*1 (sulfonamides-resistance gene), and *bla-*TEM (Extended β-lactamase gene) is conducted, in order to select the antibiotics of choice.

## 2. Results

### 2.1. Prevalence and Phenotypic Identification of E. coli

The bacteriological examination of 274 fecal swabs obtained from diarrheic calves revealed that the overall prevalence of *E. coli* was 28.8% (n = 79), based on the microscopical examination, colonial characters on MacConkey’s agar and eosin-methylene-blue agar, as well as biochemical tests. Concerning the age of the examined calves, the results revealed that 15 diarrheic calves were infected with *E. coli* in the first 2 months of age (33.3%), 41 calves at 2–4 months old (28.5%), and 23 calves at 4–6 months old (27.1%) (Table 1).

### 2.2. Serotyping of E. coli Isolates

In the present study, the serological identification of the retrieved isolates revealed that a total of 64 (81.01%) of the isolated strains were typable with O antisera, while 15 isolates (18.99%) were untypable. The most common serogroup was O128 (16.5%), followed by O111 (13.9%) and O26 (11.4%), O125 (11.4%), O91 (10.1%), O45 (8.9%), and O119 (8.9%). The frequency of *E. coli* serogroups was illustrated in Table 2. There is no statistically significant difference in the prevalence of *E. coli* serogroups (*p* > 0.05).

### 2.3. Congo Red (CR) Binding Test

The Congo red test revealed that 89.8% of the tested *E. coli* strains (n = 71) were positive, including the members of serotypes; O26, O111, O125, O128, O45, O119 and 15 untypable strains, while the strains of the O91 serogroup were CR negative (n = 8).

### 2.4. The Antimicrobial Resistance Profiles and the Antimicrobial Resistance Genes of the Isolated E. coli Strains

The antimicrobial susceptibility testing of the isolated *E. coli* strains (Table 3) showed a remarkable resistance to neomycin (96.2%), gentamycin and streptomycin (95%), and amikacin (93.7%). Furthermore, substantial sensitivity was recorded to enrofloxacin (84.9%), florfenicol (82.4%), and both amoxicillin/clavulanic acid and ampicillin/sulbactam (78.5%, each). The statistical analysis proved that the resistance of the tested strains against various antimicrobial agents was significantly different (*p* < 0.0001). Regarding the occurrence of the multidrug-resistance and the distribution of the antimicrobial resistance genes among the isolated strains, we noticed that 41.8% of the tested *E. coli* strains (n = 33) exhibited multidrug resistance to neomycin, gentamicin, streptomycin, and amikacin and harbored the *aadB* resistance gene. Moreover, 27.8% of the examined strains (n = 22) showed multidrug-resistance to neomycin, gentamicin, streptomycin, amikacin, and trimethoprim/sulfamethoxazole, and harbored both *aad*B and *sul*1 resistance genes. In addition, 21.5% of the tested strains (n = 17) exhibited a multidrug-resistance to neomycin, gentamicin, streptomycin, amikacin, amoxicillin/clavulanic acid, and ampicillin/sulbactam, and harbored both *aad*B and *bla*TEM resistance genes (Table 4).

**Figure 1:**
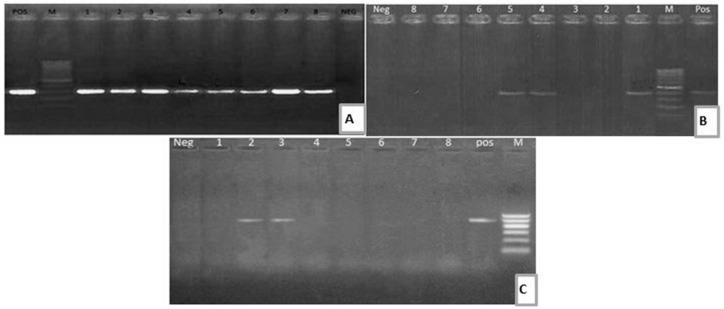
(**A**) Electrophoretic pattern of *aad*B (319 bp): M: 100–1000 bp DNA ladder; POS: Positive control; Neg: Negative control; Lanes: 1–8: positive *E. coli* strains. (**B**) Electrophoretic pattern of *sul*1 gene (433 bp): M: 100–1000 bp DNA ladder, POS: Positive control; Neg: Negative control; Lanes: 1, 4, 5: positive *E. coli* strains; Lanes: 2, 3, 6, 7, 8: negative *E. coli* strains. (**C**) Electrophoretic pattern of *bla*TEM gene (516 bp): M: 100–600 bp DNA ladder; POS: Positive control; Neg: Negative control; Lanes: 2–3: positive *E. coli* strains; Lanes: 1, 4, 5, 6, 7, 8: negative *E. coli* strains.

Concerning the distribution of the antimicrobial resistance genes; the *aad*B gene is the most predominant antimicrobial resistance gene associated with the retrieved *E. coli* strains, either alone or in combination with the *sul*1 gene (27.8%), or in combination with the *bla*TEM gene (21.5%). Moreover, the *sul*1 gene was determined alone in 8.8% of the examined strains. Statistically, there is a significant difference in the distribution of the antimicrobial resistance genes among the examined *E. coli* strains (*p* < 0.05) (Table 5 and Table 6, and Figure 1).

### 2.5. The Distribution of Virulence Genes Among the Isolated E. coli Strains

**Figure 2:**
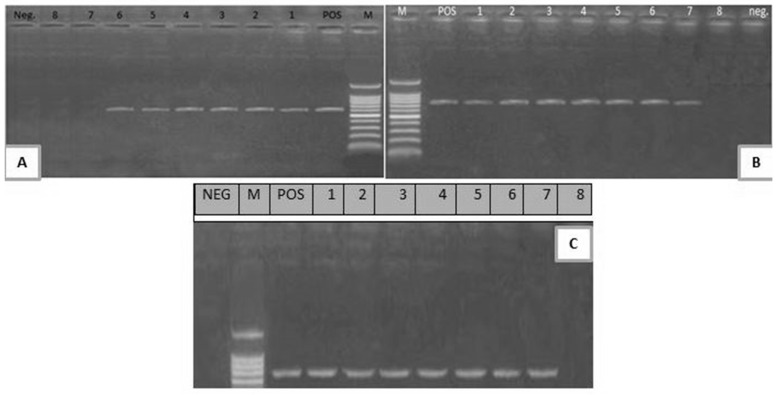
(**A**) Electrophoretic pattern of *stx*1 gene (614 bp); M: 100–1000 bp DNA ladder; POS: Positive control; Neg: Negative control; Lanes 1–6: positive *E. coli* strains; Lanes 7, 8: negative *E. coli* strains. (**B**) Electrophoretic pattern of *stx*2 gene (779 bp); M: 100–1000 bp DNA ladder, POS: Positive control; Neg: Negative control; Lanes 1–7: positive *E. coli* strains; Lane 8: negative *E. coli* strain. (**C**) Electrophoretic pattern of *eae*A gene (248 bp): M: 100–600 bp DNA ladder; POS: Positive control; NEG: Negative control; 1–7: positive *E. coli* strains; Lane 8: negative *E. coli* strain.

**Figure 3:**
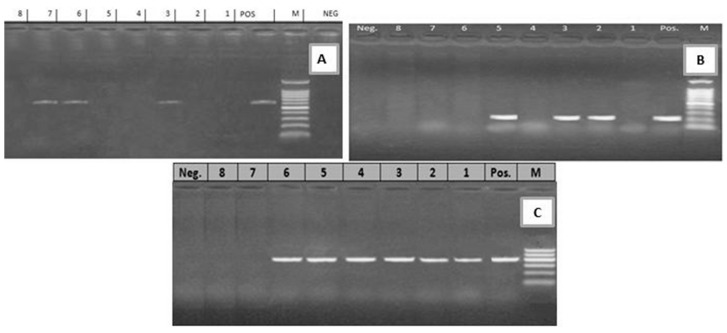
(**A**) Electrophoretic pattern of *lt* gene (605 bp): M: 100–1000 bp DNA ladder; POS: Positive control; Neg: Negative control; Lanes: 3, 6, 7: positive *E. coli* strains; Lanes 1, 2, 4, 5, 8: negative *E. coli* strains. (**B**) Electrophoretic pattern of *sta* gene (219 bp): M: 100–1000 bp DNA ladder, POS: Positive control; Neg: Negative control; Lanes: 2, 3, 5: positive *E. coli* strains; Lanes 1, 4, 6, 7, 8: negative *E. coli* strains. (**C**) Electrophoretic pattern of *f41* gene (380 bp): M: 100–600 bp DNA ladder, POS: Positive control; Neg: Negative control; Lanes: 1–6: positive *E. coli* strains; Lanes 7, 8: negative *E. coli* strains.

Regarding the distribution of the virulence genes among the examined *E. coli* strains, the PCR revealed two pathotypes (40/79, 50.6%); Shiga-toxigenic *E. coli* (STEC) (16/79, 20.2%) and Enterotoxigenic *E. coli* (24/79, 30.4%). Regarding the STEC; the *stx*1 gene is the most predominant Shiga-toxin gene, either found alone (n = 4) or in combination with *stx*2 and *eae*A genes (n = 7), or in combination with the *stx*2 gene (n = 2), while the *stx*2 gene was detected alone in three *E. coli* strains. Concerning the ETEC; the *lt* gene was the most prevalent enterotoxin gene, either found in combination with the *f*41 gene (n = 10), or in combination with *sta* gene (n = 4), or in combination with *sta* and *f*41 genes (n = 3). In addition, the *sta* gene was detected alone in seven *E. coli* strains (Table 6 and Table 7, and Figure 2 and Figure 3). There is no statistically significant difference in the distribution of virulence genes among the isolated *E. coli* strains (*p* > 0.05).

## 3. Discussion

This study was aimed at determining the prevalence of STEC and ETEC incriminated in calf diarrhea, with special reference to the Shiga-toxins genes (*stx*1 and *stx*2) and enterotoxins genes (*lt* and *sta*) that govern their pathogenesis, as well as the virulence genes; *eae*A and *f*41, and the screening of their antimicrobial resistance profiles and antimicrobial resistance genes; *aad*B, *sul*1, and *bla-*TEM. The overall prevalence of *E. coli* was 28.8%, which is lower than what was reported in diarrheic calves in Egypt (63.6%) [13], Ethiopia (36.8%) [14], Argentina (30.1%) [15] and India (85.04%) [16]; however, a lower prevalence was reported in other previous studies in Korea (22%) [17] and Switzerland (5.5%) [18]. Differences in the prevalence of *E. coli* may be due to the differences in geography, management practices, floor type, health conditions, and the calf’s age [2,13,15]. Further, the high rate of *E. coli* isolation in the present study could be attributed to many reasons, such as the mixing of different age groups, poor environmental and hygienic conditions, or the poor quantity and/or quality of colostrum. In addition, the colostrum maternal antibodies cannot neutralize the high dose of pathogenic *E. coli* infection [14].

Regarding the age of the examined calves, the prevalence of *E. coli* was 33.3%, 28.5%, and 27.1% in the first 2 months old, 2–4 months old, and 4–6 months old aged calves, respectively. There is no statistically significant difference in the prevalence of *E. coli* among the different ages (*p* = 0.74). A previous study reported that the prevalence of *E. coli* was high in the young calves, and then decreased as the age increased [14].

The serological identification of the retrieved isolates revealed that a total of 64 (81.01%) strains were typable, while 15 isolates (18.99%) were untypable. The most common serogroup was O128 (16.5%), followed by O111 (13.9%) and O26 (11.4%), O125 (11.4%), O91 (10.1%), O45 (8.9%), and O119 (8.9%). The identified serogroups in the current study have a different variety than those previously recorded [13,19,20,21], which are often associated with sick children with diarrhea [19,22].

In the current study, 89.8% of the *E. coli* strains were CR positive, including serogroups O_26_, O_111_, O_125_, O_128_, O_45_, O_119_ and 15 untypable strains; a finding endorsed by a previous study [23] stated that 95% of *E. coli* isolates are pathogenic by CR binding, which indicates the virulence of these strains. In other studies, it was reported that 61.9–90% of *E. coli* isolates were CR positive [24,25].

In the present study, the isolated strains exhibited a remarkable resistance against four antimicrobial agents, including; neomycin (96.2%), gentamycin and streptomycin (95%), and amikacin (93.7%). A previous study from Egypt in diarrheic buffalo-calves farms reported that the most prevalent phenotypic resistance patterns were; ampicillin (71.4%) and amoxicillin (64.3%), as well as trimethoprim/sulfamethoxazole (50%) and gentamicin (42.8%) [26]. A previous study in Iran reported that *E. coli* strains retrieved from diarrheic calves showed maximum resistance to penicillin, streptomycin, tetracycline, lincomycin and sulfamethoxazole [27]. The resistance pattern observed in our study and the previous other studies indicated that the emergence of multidrug-resistant species has become an increasing problem worldwide, due to the overuse of antimicrobial drugs in both animal and human medicine [28,29]. The increased pattern of multidrug resistance could be attributed to the accumulation of genes encoding for the antibiotic resistance, either on the bacterial chromosome or plasmid [12].

Concerning the occurrence of the multidrug-resistance patterns and the distribution of the antimicrobial resistance genes among the isolated *E. coli* strains, 41.8% of the tested strains showed multidrug resistance to the aminoglycosides antibiotics; neomycin, gentamicin, streptomycin, and amikacin (harbored *aad*B gene), while 27.8% exhibited multidrug resistance to the aminoglycosides antibiotics and trimethoprim/sulfamethoxazole (harbored both *aad*B and *sul*1 genes). Although clavulanic acid and sulbactam are known as effective β-lactamase inhibitors, 21.5% of the tested strains showed multidrug resistance to amoxicillin/clavulanic acid, ampicillin/sulbactam, and the aminoglycosides antibiotics (harbored both *aad*B and *bla*TEM genes). In addition, the most predominant antimicrobial resistance gene was *aad*B gene, which occurred in 72/79 of the examined strains. These findings are endorsed by a previous study, which reported that an aminoglycoside-resistance determinant (*aad*B) gene was prevalent among the pathogenic *E. coli* strains originated from diarrheic calves. Such type of resistance could be attributed to the presence of *aad*B gene, as well as the widespread improper use of aminoglycosides for the treatment of calf diarrhea in the past years [30]. Genes encoding resistance to aminoglycosides, sulfonamides, and ampicillins were *aadB*, *sul1*, and *bla*TEM genes, respectively [31]. A previous study revealed that the most usually detected β- lactamase gene is *blaTEM*, occurring in 66/74 of the tested strains; the resistance to the antimicrobial agents could be due to the presence of variable gene variants in the resistant isolates [32].

In the present study, the prevalence of the Enterotoxigenic *E. coli* was 30.4%, while the prevalence of Shiga-toxigenic *E. coli* was 20.2%. Concerning the isolated STEC strains, the *stx*1 gene is the most predominant Shiga-toxin gene, either found alone or in combination with *stx*2 and *eae*A genes, or in combination with the *stx*2 gene. A previous study from diarrheic calves in India reported that the profile of virulence genes of the STEC isolates was found in diverse combinations, and the combination of *hly*A and *eae*A genes was most the predominant [16]. Shiga toxins usually inactivate the host cell-ribosomes with subsequent inhibition of protein-biosynthesis. The occurrence of the *stx*1, *eae*A, and *stx*2 genes together constitute an epidemiological significance, as previous studies reported that the combination of these genes could increase the ability of *E. coli* to cause severe human illness [16,33].

Regarding the isolated ETEC strains, the *lt* gene was the most prevalent enterotoxin gene, either found in combination with the *f*41 gene, or in combination with the *sta* gene, or in combination with both *sta* and *f*41 genes. LT (heat-labile toxin) usually stimulates the adenylate-cyclase enzymatic system, while STa (stable-toxin) activates the guanylate-cyclase system, resulting in severe watery diarrhea. The *f*41 gene is usually associated with the occurrence of diarrhea in calves and has a higher prevalence in diarrheic calves rather than healthy ones, which warrants a great role of this gene in ETEC pathogenesis [34].

In Argentina, the most prevalent virulence genes of pathogenic *E. coli* isolated from dairy calves were *k*99, *f*41 and *f*5 [15]. In Brazil, the prevalence of the combination of *sta* and *lt* genes was 3.9% in *E. coli* isolated from diarrheic calves [35]. In China, 15.5% of the ETEC which originated from healthy calves were carried *lt* and *sta* genes [36]. Furthermore, in Italy, the *sta* gene was absent in *E. coli* that isolated from diarrheic buffalo calves [37]. The diversity in the prevalence of Shiga-toxins genes, enterotoxins genes, and other virulence-related genes in the present study and the other studies may be attributed to the geographical origin of samples, the sample size, the handling of collected samples, the number of examined strains, the type of the examined virulence genes, and the role of the examined virulence genes in the pathogenesis of the disease.

In conclusion, *E. coli* continues to be one of the major causes of calf diarrhea resulting in severe economic losses. Both Shiga-toxigenic and Enterotoxigenic *E. coli* are the most prevalent pathotypes incriminated in the disease occurrence. The Shiga toxins genes; *stx*1 and *stx*2, are the most prevalent virulence genes associated with STEC, which are responsible for the pathogenesis of the disease and helped by the intimin gene (*eae*A). Furthermore, the *lt* gene is the most prevalent enterotoxin gene which accompanies the ETEC strains isolated from calf diarrhea, either alone or in combination with the *sta* and or the *f*41 genes. The majority of the isolated STEC and ETEC harbored *aad*B antibiotic resistance gene and exhibited a multidrug-resistance pattern to neomycin, gentamycin, streptomycin, and amikacin. Moreover, Enrofloxacin, florfenicol, amoxicillin-clavulanic acid, and ampicillin-sulbactam are the most effective antimicrobial agents against the isolated STEC and ETEC strains. The incessant implementation of the antibiogram profile is needed to determine the most effective antibiotic, due to the high prevalence of the multidrug-resistant *E. coli* strains.

## 4. Materials and Methods

### 4.1. Sampling

A total of 274 fecal specimens were aseptically gathered using sterile rectal swabs from April 2018–Feb 2019, from diarrheic calves aged between 1 day and 6 months, from two private farms (124 calves from farm I and 150 from farm II) at El-Sharqia governorate, Egypt. The collected specimens were rapidly transferred to the bacteriological lab, Faculty of Veterinary Medicine, Suez Canal University, for further analysis. The handling of animals was carried out by well-trained scientists, according to the instructions of the Animal Ethics Review Committee of Suez Canal University (SCU-362-27/12/2016).

### 4.2. Isolation and Identification of E. coli

The collected specimens were inoculated in nutrient broth and then incubated for 24 h at 37 °C. Loopful from the culture suspension was streaked on MacConkey’s agar and Eosin methylene blue agar (Oxoid, UK). The recovered typical colonies (pink colonies on MacConkey’s agar and metallic green sheen colonies on EMB) were completely identified morphologically and biochemically, as described by Quinn et al. [38].

### 4.3. Serotyping of Isolated E. coli

The obtained *E. coli* isolates were serogrouped by the detection of O antigens using the slide agglutination test, according to the method previously described by Edwards and Ewing [39].

### 4.4. Congo Red Test

The Congo red (CR) binding test has been used to detect the invasive *E. coli*, using trypticase agar supplemented with 0.03% CR dye (Oxoid, UK). The colonies were streaked on Congo red agar and incubated at 37 °C for 24 hrs, then plates were kept at room temperature for 48 hrs. The demonstration of the invasive strains (red colonies) was observed and recorded according to the methods previously described by Panigrahy and Yushen [24].

### 4.5. Antimicrobial Susceptibility Testing

The isolated *E. coli* strains were tested against nine antimicrobial agents; amoxicillin/clavulanic acid (20/10 mcg), ampicillin/sulbactam (10/10 mcg), amikacin (10 mcg), neomycin (30 mcg), enrofloxacin (5 mcg), florfenicol (30 mcg), streptomycin (10 mcg), trimethoprim/sulfamethoxazole (25 mcg) and gentamicin (30 mcg) (Oxoid, UK), using the disc diffusion method. The diameter of the inhibition zone was measured in millimeters and expressed as sensitive, intermediate, and resistant, as described by CLSI [40].

### 4.6. PCR Based Detection of Virulence Genes and Antmicrobial Resistance Genes

Bacterial DNA of purified bacterial cells was extracted using the QIAamp DNA Mini Kit (Invitrogen, USA). Recovered DNA templates were quantified using a Nanodrop (Nanodrop 1000, Thermo Scientific, Loughborough, UK), adjusted to 100 ng μL^−1^. To assess the virulence genes (*stx*1, *stx*2 *lt*, *sta*, *f*41 and *eae*A), as well as the antimicrobial resistance genes (*aad*B, *sul1*, and *bla*-TEM) in the obtained *E. coli* strains, PCR was performed using specific sets of primers (Metabion, Germany) (Table 8). The PCR reaction (25 μL) consists of 12.5 μL Go Taq^®^ Green Master Mix 2X (Promega, Wisconsin, USA), 1 μL (20 pmol) of each primer, 5 μL DNA extract, and PCR grade water up to 25 μL. The cycling conditions are listed in Table 8. Negative control (no DNA template) and positive control reference strains (previously isolated and kindly provided by A.H.R I, Dokki, Egypt) were used in the PCR assay. Amplified fragments were screened by 1.5% agarose gel electrophoresis (Applichem GmbH, Darmstadt, Germany) for 45 min at 100 V in 1× TAE, visualized using 15 µL of DNA gel stain (Sigma-Aldrich, St. Louis, MI, USA) and photographed under UV transilluminator. A 100 bp ladder (Fermentas, Thermo Scientific, Darmstadt, Germany) was used.

### 4.7. Statistical Analysis

The Chi-square was performed to analyze the data, to test the null hypothesis of different treatments using the statistical analysis software (SAS^®^ software version 9.4, SAS Institute, Cary, NC, USA). The significance level was (*p <* 0.05).

## Figures and Tables

**Table 1:** Prevalence of pathogenic *E. coli* in diarrheic calves at different ages.

Ages (Months)	Number of Samples	No. of *E. coli*	Prevalence (%)
1–2 days	45	15	33.3
2–4	144	41	28.5
4–6	85	23	27.1
Total	274	79	28.8

*p* value = 0.74 (Not significant; *p* > 0.05).

**Table 2:** Frequency of *E. coli* serogroups in diarrheic calves.

Serotypes		Number (%)
O 128		13 (16.5)
O 111		11(13.9)
O 125		9 (11.4)
O 26		9 (11.4)
O 91		8 (10.1)
O 45		7 (8.9)
O 119		7 (8.9)
Total	Typable	64 (81.01)
	Untypable	15 (18.99)

*p* value = 0.73 (Not significant; *p* > 0.05).

**Table 3:** In Vitro susceptibility pattern of the isolated *E. coli* strains (n = 79) against different antimicrobial agents.

Antibiotics	Sensitive		Intermediate		Resistant	
	No.	%	No.	%	No.	%
**Amoxicillin/Clavulanic acid**	62	78.5	0	0	17	21.5
**Ampicillin/Sulbactam**	62	78.5	0	0	17	21.5
**Enrofloxacin**	67	84.9	7	8.8	5	6.3
**Trimethoprim/Sulfamethoxazole**	30	38	20	25.3	29	36.7
**Gentamycin**	0	0	4	5	75	95
**Neomycin**	0	0	3	3.8	76	96.2
**Florfenicol**	65	82.4	10	12.6	4	5
**Streptomycin**	0	0	4	5	75	95
**Amikacin**	0	0	5	6.3	74	93.7
**Chi-Square value** ***p* value**	442.4663 *p* < 0.0001		56.4924 *p* < 0.0001		468.007 *p* < 0.0001	

**Table 4:** The distribution of the multidrug resistance patterns and the antimicrobial resistance genes among the tested *E. coli* strains (n = 79).

No of Isolates	% of Isolates	The Multidrug Resistance Patterns	The Antimicrobial Resistance Genes
33	41.8	Neomycin, gentamicin, streptomycin, and amikacin	*aad*B
22	27.8	Neomycin, gentamicin, streptomycin, amikacin, and trimethoprim/sulfamethoxazole	*aad*B, *sul*1
17	21.5	Neomycin, gentamicin, streptomycin, amikacin, amoxicillin/clavulanic acid, and ampicillin/sulbactam	*aad*B, *bla*TEM
3	3.8	Neomycin, gentamicin, streptomycin, trimethoprim/sulfamethoxazole, enrofloxacin, and florfenicol	*sul*1
2	2.5	Amikacin, trimethoprim/sulfamethoxazole, and enrofloxacin	*sul*1
1	1.3	Neomycin, trimethoprim/sulfamethoxazole, and florfenicol	*sul*1
1	1.3	Trimethoprim/sulfamethoxazole	*sul*1

**Table 5:** The distribution of antimicrobial resistance genes in *E. coli* strains isolated from diarrheic calves (n = 79).

Antimicrobial Resistance Genes	*E. coli*	
	No	%
***sul*1, *aad*B**	22	27.8
***aad*B**	18	22.7
***aad*B, *bla*TEM**	17	21.5
***sul*1**	7	8.8
***aad*B**	15 (untypable strains)	19

*p* value = 0.04 (Significant; *p* < 0.05).

**Table 6:** The distribution of virulence and antimicrobial resistance genes among the isolated *E. coli* serotypes.

Target Genes		O 128 n = 13	O 111 n = 11	O 125 n = 9	O 26 n = 9	O 91 n = 8	O 45 n = 7	O 119 n = 7	Untypable n = 15	Total
**Virulence genes**	***stx*1**	1	1	0	1	1	0	0	0	4
	***stx*2**	1	1	0	1	0	0	0	0	3
	***stx*1 + *stx*2**	1	0	0	1	0	0	0	0	2
	***stx*1 + *stx*2 + *eae*A**	2	1	1	1	1	0	1	0	7
	***lt*, *f*41**	1	1	1	2	2	2	1	0	10
	***sta***	1	0	2	1	1	1	1	0	7
	***lt*, *sta***	1	0	1	1	0	0	1	0	4
	***lt*, *f*41, *sta***	1	0	0	1	0	0	1	0	3
	**Total**	9	4	5	9	5	3	5	0	40
**Antibiotic resistance genes**	***sul*1, *aad*B**	6	4	4	4	1	1	2	0	22
	***aad*B**	3	3	2	3	2	2	3	15	33
	***aad*B, *bla*TEM**	3	4	2	1	3	3	1	0	17
	***sul*1**	1	0	1	1	2	1	1	0	7
	**Total**	13	11	9	9	8	7	7	15	79

**Table 7:** The prevalence of the virulence genes in *E. coli* strains isolated from diarrheic calves (n = 79).

Pathogenic *E. coli*	Virulence Genes	*E. coli*		*p*-Value
		No	%	
**Shiga-toxigenic *E. coli* (STEC)**	*stx*1	4	5	0.19 ^NS^
	*stx*2	3	3.7	
	*stx*1, *stx*2	2	2.5	
	*stx*1, *stx*2, *eae*A	7	8.9	
**Sub-total**		16	20.2	
**Enterotoxigenic *E. coli* (ETEC)**	*lt*, *f*41	10	12.7	0.09 ^NS^
	*sta*	7	8.9	
	*lt*, *sta*	4	5	
	*lt*, *f*41, *sta*	3	3.7	
**Sub-total**		24	30.4	
**Total**		40	50.6	

NS = Not significant.

**Table 8:** Primers sequences and recycling conditions of PCR assays used for the detection of virulence genes and antimicrobial resistance genes.

Target Gene	Oligonucleotide Sequence (5′-3′)	Product Size (bp)	Denat.	Cycling (35)			Final E	References
				D	A	E		
*eae*A	F: GTGGCGAATACTGGCGAGACT R: CCCCATTCTTTTTCACCGTCG	248	95 °C 5 min	95 °C 30 s	60 °C 30 s	72 °C 30 s	72 °C 10 min	[41]
*stx*1	F: ACACTGGATGATCTCAGTGG R: CTGAATCCCCCTCCATTATG	614	95 °C 5 min	95 °C 3 min	59 °C 45 s	72 °C 90 s	72 °C 10 min	[42]
*stx*2	F: CCATGACAACGGACAGCAGTT R: CCTGTCAACTGAGCAGCACTTTG	779	95 °C 5 min	95 °C 3 min	59 °C 45 s	72 °C 90 s	72 °C 10 min	[42]
*sta*	F: GAAACAACATGACGGGAGGT R: GCACAGGCAGGATTACAACA	229	94 °C 5 min	94 °C 30 s	57 °C 45 s	72 °C 45 s	72 °C 10 min	[43]
*lt*	F: GGTTTCTGCGTTAGGTGGAA R: GGGACTTCGACCTGAAATGT	605	94 °C 5 min	94 °C 30 s	57 °C 45 s	72 °C 45 s	72 °C 10 min	[43]
*f*41	F: GCATCAGCGGCAGTATCT R: GTCCCTAGCTCAGTATTATCACCT	380	94 °C 5 min	94 °C 30 s	50 °C 45 s	72 °C 90 s	72 °C 10 min	[44]
*aad*B	F: GAGCGAAATCTGCCGCTCTGG R: CTGTTACAACGGACTGGCCGC	319	94 °C 5 min	94 °C 30 s	55 °C 45 s	72 °C 45 s	72 °C 10 min	[45]
*bla*TEM	F: ATCAGCAATAAACCAGC R: CCCCGAAGAAC GTTTTC	516	94 °C 5 min	94 °C 30 s	55 °C 45 s	72 °C 45 s	72 °C 10 min	[46]
*sul*1	F: CGGCGTGGGCTACCTGAACG R: GCCGATCGCGTGAAGTTCCG	433	94 °C 5 min	94 °C 30 s	55 °C 45 s	72 °C 45 s	72 °C 10 min	[47]

D, Denaturation; A, Annealing; E, Extension.
